# A complementary response to the article “breaching confidentiality: medical mandatory reporting laws in Iran”

**Published:** 2015-11-10

**Authors:** Omid Asemani

**Affiliations:** ^1^Assistant Professor, Department of Medical Ethics and Philosophy of Health, Faculty of Medicine, Shiraz University of Medical Sciences, Shiraz, Iran.

Enthusiastically, I read the article published in the issue 2014 (1) well addressed an abiding conflict between “professional confidentiality” and “mandatory reporting laws”. In the following have been tried to provide a brief explanation in purpose of introducing the nature of the conflict in a more comprehensive view. In this way, I have discussed why medical confidentiality needs to be protected completely unless formal laws make members of the health care team to divulge minimum professional information to specific legal authorities. In this way, I have described that every society, as a multi-individual organism, needs to defend its integrity against individual's harmful (not legitimate) desires and actions through enforcing laws and regulations.

At the beginning, it is important to clarify, or better say, remind that one of the main consequences or I see, purposes of respecting confidentiality in medical practice is bolstering people confidence in health care providers. Knowingly, trust is a critical prerequisite of medical practice; therefore, the critical role of confidentiality in building a safe medical environment and thus providing a good medical care is incontrovertible. In this way, bolstering trust in the physician-patient relationship via emphasizing on a complete medical confidentiality could overall improve public health for at least two main reasons: patients could willingly seek medical attention and then give doctors authentic information they need to provide a good care. Surely, lack of professional commitment on confidentiality would not guarantee a secure atmosphere for patients to be treated free from any concern to get stuck in undesired and unpredicted circumstances (2-4). 

In spite of this importance, oppositely, there are circumstances in which protecting absolute confidentiality of an individual does not bring about constructive consequences for (all) others. For example, practically, complete confidentiality in cases of murder, child abuse and so on clearly contradicts public health, social security and social responsibility. In fact, in these conflicting situations, confidentiality might practically run counter to those positive outcomes (trust related outcomes) expected to be achieved when preserving it in an absolute way. Overall, medical confidentiality might result in two kinds of individual and public consequences, those that not only might not be necessarily in line with each other but also opposing. Accordingly, a complete protection of confidentiality for an individual might be harmful for another individual or the public in short and especially long terms. Certainly, the conflict could not be logically dealt with in the medical field, because logically, the profession itself cannot be synchronously the source of two conflicting things: to recommend absolutism on one side and to determine breaking instances on the other side.

Generally, we need to solve conflicts as soon and precise as possible for they fritter away our limited time, energy and money. But, other than the logic of the issue, medical professionalism should not determine breaking criteria or instances by itself for at least two trust-related reasons; firstly, for the fundamental importance of building a trusty relationship between patients and health care team and its critical role in preserving and improving public health especially in long terms; and secondly, for destructive effects of losing the trust in endangering public health and security (2) and also its –trust- really hard and lengthy reconstruction to the level that could works constructively in social reactions. For these reasons, we ought to approach the conflict from outside of medicine for minimizing disadvantages. In this respect, law, I think, has been the best case for having three main characteristics all suitable for mediating negative outcomes of breaching medical confidentiality: its concern for justice, extensive public acceptance and having executive power; applying these positive potentials, we could hope that destructive effects of breaking confidentiality, especially on the trust, to be dealt with to a minimum.

Otherwise, absolutism in medical confidentiality could jeopardize "public autonomy" in both short and long terms. Here, "public autonomy" can be considered as a shared autonomy by which members of a society agree to respect it as "a common interest"; so that their personal autonomies would be confined within the boundaries of the public as long as they live or attend that society. Usually, regulations, laws and legal authorities have charge of protecting and supporting the "public autonomy". As a general rule of social living, no personal action or desire should neglect “public interest”; otherwise, legal authorities defend the “public autonomy” and counteract it using laws and legal forces. This is true about all social structures and establishments including medicine. Therefore, personal irresponsibility and intemperate and self-centered behavior of individuals that might harm others could not be socially disregarded even under the coverage of professional confidentiality. As aforementioned, in these instances, law, because of its specific characteristics, has been the best case for making conditional exceptions of medical confidentiality with the least negative effects.

In sum, I believe we, members of the health care team, are strongly in need of maintaining a total confidentiality for the critical matter of trust and its profound effects on the public health and security. On the other hand, we are also required to obey "mandatory reporting laws" as a necessary requirement of social living and as an efficient mean in controlling harmful and anti-social behavior of individuals. Nevertheless, in line with the professional confidentiality, our first-line duty is basically to spend enough time and energy for solving conflicting cases within the boundaries of the profession (to observe a complete confidentiality) unless someone wants to pursue his/her socially harmful decision or behavior obstinately which then should be managed using related laws.
